# Measuring the Impact of Integrated Care: from Principles to Real-World Impact

**DOI:** 10.5334/ijic.7783

**Published:** 2023-11-29

**Authors:** Jessica Michgelsen, Nick Zonneveld, Ephrem Tesfay, Mirella Minkman

**Affiliations:** 1 Vilans, National Centre of Expertise in Long Term Care, Utrecht, The Netherlands; 2 GGD Haaglanden, The Netherlands; 3 Tilburg University, Department of Organization Studies, The Netherlands; 4 Tilburg University-TIAS Business school, The Netherlands

**Keywords:** impact, integrated care, measurement, Theory of Change

## From principles to real-world impact

Integrated care has been recognized as a global movement in transforming care systems to promote person centred care, and to accommodate the growing care demand amidst the rapidly changing and ageing global population [1,2]. The scope of its practical implementation and the intended impact of integrated care can be wide and diverse. For example, integrated care can be seen to improve the coordination of care or ensure streamlined care pathways; to reduce unnecessary use of institutional care; to reduce costs; to improve people’s care experiences and outcomes; to support workforce developments; to improve public health and even to address inequalities in care. This wide range of objectives brings along numerous complexities to be able to assess the impact of integrated care. It requires a multifaceted, methodologically robust approach that considers the whole spectrum of operationalised integrated care and balancing impact across various aspects and stakeholders [3,4,5].

To gain insights into the contemporary landscape of integrated care’s impact, an international team of researchers conducted a comprehensive analysis of recently published IJIC articles to explore the extent to which authors intend to assess the impact of integrated care. For this ambitious aim, an analysis based on an ‘all inclusive’ comprehensive framework that captures the impact of the integrated care service(s) would be ideal. If possible, it would resonate with the viewpoints of all involved stakeholders. The team concluded that such a framework was lacking and applied the widely recognized quadruple aim model [6] that highlights service user experience, staff experience, costs of care, and health outcomes as the most important categories for impact. Nevertheless, despite employing a predefined model, achieving consensus within the research group on the precise boundaries of what exactly constitutes impact and what falls outside of its scope proved to be elusive [paper yet to be published].

Hence, in this editorial, we navigate through three of the complexities that seem to come at play when measuring the impact of integrated care. Concurrently, we invite further academic discussion and look forward to welcoming submissions of papers to IJIC’s special collection focusing on innovative methods that measure impact of integrated care in current contexts. The call for papers will soon be announced.

## Complexity 1: Identifying aspects to assess impact

Conceptual models showing principles of integrated care, such as IFIC’s knowledge tree [7], the Development Model of Integrated Care [8] or the conceptual model for Integrated Community Care [9], all demonstrate a range of integrated care principles and features. Exploring the impact of integrated care demands a comprehensive examination of these core principles and features, and their tangible implementation within care interventions. Several issues emerge when we look into the alignment of integrated care’s principles and features with the measurable objectives that can define impact. To what extent have the principles been applied? Have specific features and elements been singled out for implementation, or have they been employed in their entirety? The way these principles are utilized fundamentally shapes the perspective from which we perceive impact.

For instance, Lewis et al. evaluated a decade of three integrated care pilot programmes of the National Health System in England which aimed for better coordination between hospital and community-based health services. Despite positive experiences from involved staff, there was little shared understanding of the concept of integrated care, which led among national sponsors to increasing focus on a single outcome measure, namely reducing unplanned hospital admissions. The pilot’s impact on unplanned admissions however was limited, and therefore the added value of the integrated care approach remained subject of debate [10]. The lack of consensus among policy makers and local health teams about the definition of integrated care and the pilot’s objectives hampered the successive programs to effectively learn from each other.

## Complexity 2: Methodological challenges

Once the aspects are identified that should be minimally measured to assess impact, researchers can face a number of methodological challenges. One of the major challenges is to develop a comprehensive tool that captures the complex dynamics and multiple layers of integrated care implementation, in a context that may change over time [11].

As such, integrated care interventions seem less evident when methodologies fail short in capturing the ongoing nature of impact beyond the intervention time frame. This may be rooted in fundings that predominantly endorse project spans with an average duration of 2-5 years. This temporal constraint appears notably inadequate when considering the intricate nature of measuring impact in integrated care contexts. Some aspects of impact could be apparent in the timeline defined within the projects, while others need more time to fully materialise. Particularly in light of any transitional period averaging 10-15 years [12], it becomes evident that current project durations seem insufficient. Thus, there arises a compelling need for sustained, long-term follow-up evaluations that align with processes of either negative or positive impactful change within integrated care. During these time spans, contexts also change which further complicates the eventual reflection on the impact that can be found. Besides, due to the considerable diversity in the implementation of integrated care in practice, it has proven to be challenging to ascertain which activities or interventions have led to which type of impact.

Theory of Change (ToC) may be seen as a useful approach for understanding how interventions lead to impact [13]. Unlike simple logic models, ToC explores underlying assumptions and contextual factors, clarifying the intricate interconnections between interventions and impact, and is therefore particularly valuable for integrated care [14]. However, even with well-structured and comprehensive frameworks like the ToC, ‘real-world’ research challenges come at hand. For instance, in the case of the *InCare Project* [15,16], in one of the countries the data collection was not sufficiently coordinated among the different stakeholders, leading to a delay in showing impact of the project. This situation also carries the risk that individuals unaccustomed to researchers’ timeframes (which can span years before showing effects) may disengage from the project, despite the significance of their participation in providing diverse perspectives and interests beyond those of researchers. However, working from a ToC framework can be supportive to define impact measures at specific levels in specific contexts, and show how they contribute to the level of impact that is eventually aimed for. The drawback is the highly context specific nature of such ToC frameworks which again complexifies having a single collective understanding of the impact on integrated care.

## Complexity 3: Different viewpoints and values

Even with a clear understanding of the specific aspects to measure in integrated care and appropriate measurement tools, navigating diverse people-stakeholder perspectives remains a challenge. Integrated care often means something different for different people in different circumstances [17,18]. Care receivers, caregivers, and policy makers interpret and value integrated care impact differently based on their unique viewpoints [19]. A recent European study on stakeholders’ values in integrated care highlighted significant variations. While all groups agreed on 18 overarching integrated care values, service users and informal carers emphasised care experience values like respect and trust, while policy makers prioritised governance and organisational values such as coordination and accountability [20].

Besides the diversity in viewpoints and the multiple dimensions of integrated care, the context and cultural values in which care interventions are evaluated shape its interpretation across different care settings. For example, in countries with a more communal culture, integrated care could have a strong community-based approach, while individualistic societies could interpret integrated care as a more autonomous person-centred approach. Consequently, evaluating impact of integrated care necessitates context specific framing and valuing rather than adapting a one-size-fits-all concept of integrated care.

It is important to acknowledge that various values and viewpoints are all potentially valid. This implies that the concept of integrated care should not be narrowly constrained by a single definition or viewpoint, but has to be regarded as a broad, encompassing term. This, however, also means that the assessment of integrated care’s impact is a multifaceted endeavour, heavily reliant on the subjective lens through which it is examined. One might be able to capture these subjective lenses and create some alignment of the overarching impact that is aimed for by employing co-creation principles. Unfortunately, co-creation does not seem to be an often-applied approach yet in the field of integrated care research despite its opportunities to give a voice to people who do not automatically have one [21]. Thus, even in an ostensibly optimal scenario, the intricate process of assessing impact necessitates careful considerations and involvement of different people.

## Conclusion

In summary, our analysis and discussion highlight the complexity of moving from principles of integrated care to measuring real life impact. Our arguments emphasise the importance of 1) identifying what impact means to different stakeholders, 2) employing appropriate tools and a Theory of Change to capture different aspects of impact and involving key stakeholders in this process, and 3) ensuring a sufficiently (extended) timeframe for a comprehensive assessment of integrated care impact.

Even in an optimal scenario, the challenge persists: how to reconcile the diverse perspectives and values of key stakeholders? This dilemma leads us to a vital question: how can we effectively evaluate and prioritise these varied viewpoints and values when gauging the impact of the integrated care service in question? Therefore, we will consistently need to make deliberate but subjective decisions about which aspects to prioritise when measuring impact. These choices, in return, shape the extent of impact we can grasp.
